# Long‐Term or Recurrent Antibiotic Use in Early Life and the Risk of Type 2 Diabetes: A Population‐Based Prospective Cohort and a Case–Control Study

**DOI:** 10.1111/1753-0407.70113

**Published:** 2025-06-18

**Authors:** Zijun Li, Qiangsheng He, Xin He, Xin Xing, Songbo Fu, Xiaoping Sun, Mina Ma, Danni Wang, Ningning Mi, Jinyu Zhao, Jinqiu Yuan, Kehu Yang

**Affiliations:** ^1^ Evidence‐Based Social Science Research Center/Health Technology Assessment Center, School of Public Health Lanzhou University Lanzhou China; ^2^ Key Laboratory of Evidence‐Based Medicine and Knowledge Translation of Gansu Province Lanzhou University Lanzhou China; ^3^ Department of Epidemiology and Biostatistics, Clinical Big Data Research Center, the Seventh Affiliated Hospital Sun Yat‐Sen University Shenzhen China; ^4^ Chinese Health RIsk MAnagement Collaboration (CHRIMAC) Shenzhen China; ^5^ Evidence‐Based Medicine Center, School of Basic Medical Sciences Lanzhou University Lanzhou China; ^6^ Department of Endocrinology The First Hospital of Lanzhou University Lanzhou China; ^7^ Health Management Center The Second Hospital of Lanzhou University Lanzhou China; ^8^ The First School of Clinical Medicine Lanzhou University Lanzhou China

**Keywords:** antibiotics, case–control study, cohort study, type 2 diabetes, UK biobank

## Abstract

**Background:**

Antibiotics in childhood are commonly used and have been linked to gut microbiome dysbiosis and metabolic disorders. However, direct evidence regarding the association between long‐term or recurrent antibiotic use (LRAU) during early life and diabetes was scarce. We performed this study to investigate this association in two population‐based studies.

**Methods:**

We undertook a prospective analysis encompassing 147 010 participants from the UK Biobank. Cox proportional hazard regression was used to calculate hazard ratios (HRs) and 95% confidence intervals (CIs) of self‐reported LRAU during early life on diabetes risk. We also conducted a case–control study within the Chinese population, in which 263 diabetes cases and 526 controls were matched for age and living location. Odds ratios (ORs) and 95% CI were was calculated using logistic regression models.

**Results:**

We identified 4314 incident cases of type 2 diabetes over 1 840 944 person‐years of follow‐up in the UK Biobank. LRAU during early life was associated with a 26% higher risk of diabetes after accounting for putative risk factors (HR, 1.26; 95% CI, 1.16–1.37) in the UK biobank. We observed a more evident association between LRAU and an elevated risk of diabetes in the case–control study (OR, 3.32; 95% CI, 2.06–5.38). The primary finding was robust to several subgroup analyses and sensitivity analyses.

**Conclusions:**

LRAU during early life may increase the risk of type 2 diabetes. Caution should be exercised when prescribing long‐term or recurrent antibiotics to children and adolescents.

## Graphical abstract

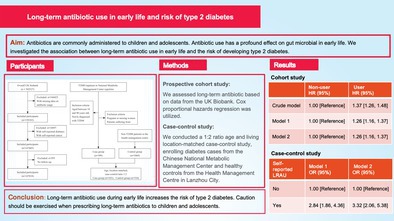

**Summary**

- Our prospective cohort study using data from the UK Biobank revealed that long‐term or recurrent antibiotic use in early life is associated with a 26% increased risk of developing type 2 diabetes.
- Similarly, a matched case–control study conducted in China also identify a heightened risk of type 2 diabetes associated with antibiotic use during early life.
- These findings underscore the importance of exercising caution when considering prolonged antibiotic therapy for children and adolescents.

## 1 Introduction

Diabetes poses a major global health threat, causing life‐threatening, disabling and costly complications and reducing life expectancy [1]. The estimated global prevalence of diabetes reached 8.5% in 2014 and is expected to rise to 12.2% by 2045, affecting almost 783 million people worldwide [2, 3]. Type 2 diabetes is the most common type of diabetes, accounting for more than 90% of cases [4]. The pathogenesis of type 2 diabetes involves a combination of genetic predisposition, lifestyle choices, and environmental influences [5]. Accumulating evidence indicates that gut microbial dysbiosis has a close relationship with type 2 diabetes progression [6]. Certain drugs, such as proton pump inhibitors and antibiotics, have the potential to mediate perturbation of the gut microbiome [7, 8].

Antibiotics, as one of the most commonly prescribed medications, have revolutionized modern medicine and extended the average human lifespan in the past century [9]. However, the widespread prescription of antibiotics has raised concerns for public health. Between 2000 and 2010, the prescription of antibiotic drugs increased by 35%, and worldwide consumption reached 70 440 786 553 standard units in 2010 [10]. This situation is particularly concerning in developing countries, where rising incomes enable greater access to antibiotics [10, 11]. As a developing country, China reported that up to 50% of prescriptions in secondary and tertiary hospitals consist of inappropriate antibiotic prescriptions [12]. Antibiotic use can disrupt the human microbiome and has been associated with chronic metabolic diseases, particularly obesity and diabetes [13, 14].

Several cohort and case–control studies have demonstrated an association between antibiotic use and an increased risk of type 2 diabetes [15, 16]. A cohort study showed that self‐reported long‐term or recurrent antibiotic use (LRAU) over the past 4 years was associated with a 20% higher risk of type 2 diabetes in women [16]. However, existing studies primarily focused on antibiotic exposure in adults and considered recent exposure, leaving a research gap regarding the potential effect of antibiotic overuse in early life. Children and adolescents are vulnerable to frequent and substantial exposure to antibiotics [17, 18]. Widespread antibiotic therapy is common for respiratory infections in childhood and acne in adolescence [19]. Early‐life events could influence gut microbiota, significantly disrupting the ecological balance of the gut microbiome with a reduction in richness and diversity, as well as alterations in the balance of beneficial commensal bacteria, which may contribute to disease progression [20, 21].

Given the limited evidence concerning the influence of LRAU in early life on the development of type 2 diabetes, this study aims to investigate this association based on the UK Biobank. Additionally, we designed a case–control study within the Chinese population with a different genetic background.

## 2 Methods

### 2.1 UK Biobank Cohort

#### 2.1.1 Study Design and Population

**FIGURE 1:**
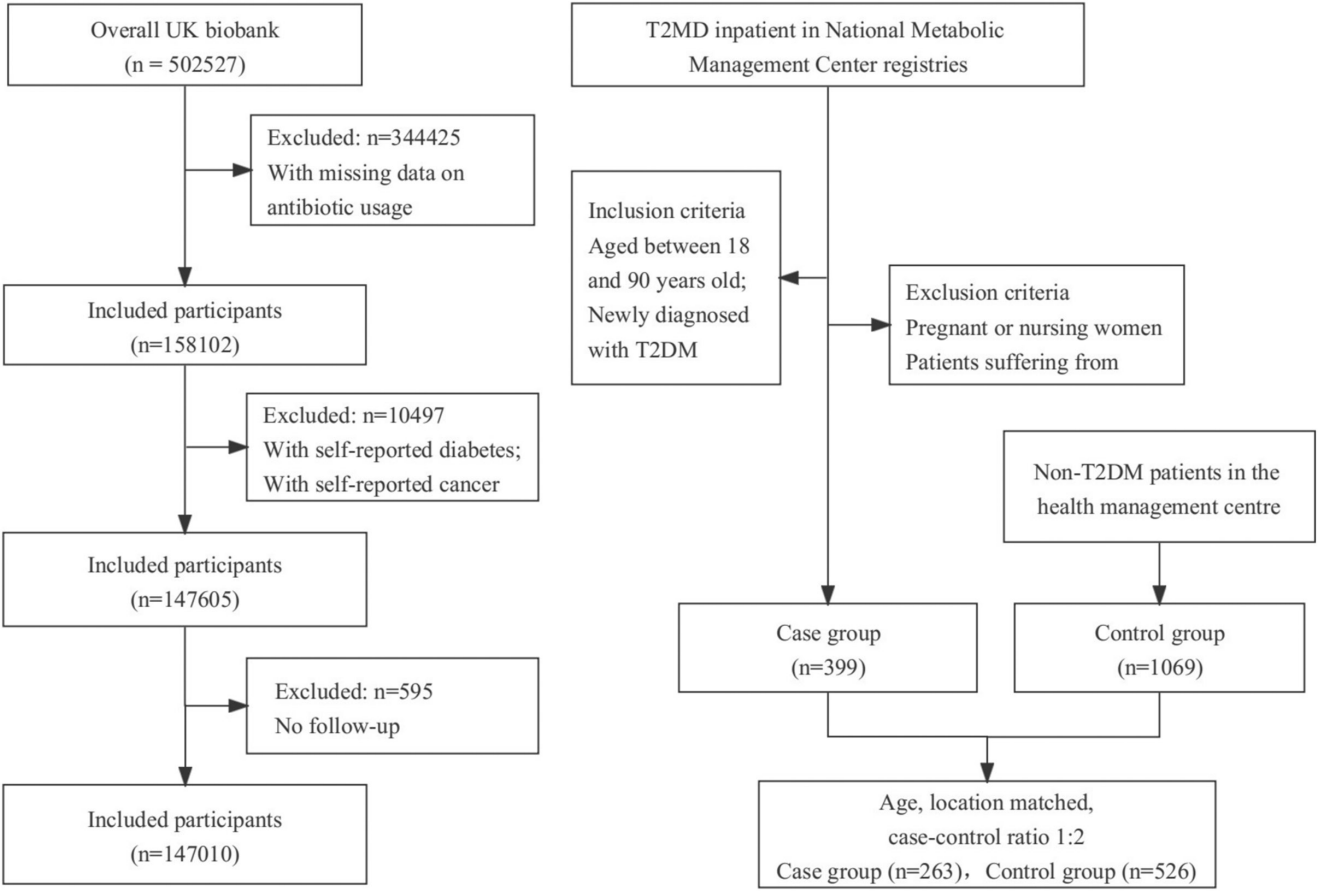
Flowchart of participants eligible.

We conducted a prospective cohort study among participants enrolled in the UK Biobank. The UK Biobank is a population‐based prospective cohort study that recruited over 0.5 million participants between 2006 and 2010, aged 40–69. Participants completed questionnaires, interviews, and physical and functional measurements. Samples of blood, urine, and saliva were obtained for subsequent laboratory analysis [22]. For this study, we included participants with detailed information on antibiotic use during childhood and adolescence (*n* = 158 102). After exclusions of participants with a preexisting diagnosis of diabetes (*n* = 3414) or cancer (*n* = 7244), a total of 147 605 participants met the inclusion criteria at baseline. Participants were followed until the first diagnosis of diabetes or censoring (December 2021). Missing data with self‐reported antibiotic use habits in early life were excluded, resulting in a final sample of 147 010 participants (Figure 1). Ethical approval for the UK Biobank was obtained from the Northwest Multi‐Centre Research Ethics Committee, and all participants provided consent. The application reference number was 51 671, approved in August 2019.

#### 2.1.2 Ascertainment of Outcome

The primary outcome of this study was the incidence of type 2 diabetes cases, which was defined by ICD‐10 codes E11. The UK Biobank obtained access to hospital inpatient records that included information on admissions and diagnoses sourced from the Hospital Episode Statistics for England, Scottish Morbidity Record data for Scotland, and the Patients Episode Database for Wales [23].

#### 2.1.3 Ascertainment of Exposure and Covariates

The term “long‐term or recurrent antibiotic use” in the questionnaire refers to the repetitive or recurrent administration of antibiotics. Participants were asked whether they received long‐term or recurrent courses (three or more per year) of antibiotics (e.g., for tonsillitis or acne) during childhood or adolescence, and participants could answer “Yes” or “No”.

The covariates were determined from baseline questionnaires and physical examinations, including personal information on age, sex, ethnicity, index of multiple deprivations (IMDs), body mass index (BMI), comorbidities (hypertension, hyperlipidemia, cardiovascular disease), drinking frequency, smoking frequency, physical activity, fruit/meat intake, and family history of diabetes. Additionally, the UK Biobank provided information on medication usage (statin, aspirin, nonsteroidal anti‐inflammatory drug [NSAID], steroids).

### 2.2 Case–Control Study

#### 2.2.1 Study Design and Population

We conducted a case–control study in the Chinese population. Type 2 diabetes patients were enrolled at the Chinese Metabolic Management Center (MMC) of the First Hospital of Lanzhou University. MMC is a new metabolic disease management model that has established an independent digital medical record system integrating real‐time in and out of hospital information of patients into a centralized medical data center [24]. Type 2 diabetes cases from the MMC were confirmed and classified based on the World Health Organization criteria [25]. Telephone interviews were conducted with newly diagnosed type 2 diabetes patients in 2021 and 2022 at the MMC.

Controls were healthy individuals without a diabetes diagnosis who were participating in physical examinations at the Health Management Centre of the Second Hospital of Lanzhou University. We matched cases and controls based on age and living location in early life (rural or urban area) at a 1:2 ratio. After excluding ineligible cases, the study population included 399 cases and 1069 controls. After matching, there were 263 cases and 526 controls (Figure 1). All participants provided informed consent. Ethical approval was obtained from the Ethics Committee of the Public Health College, Lanzhou University, China (number IRB#23062801). The study was registered with the Chinese Clinical Trial Registry. The registration number was ChiCTR2300073133.

#### 2.2.2 Ascertainment of Exposure and Covariates

Antibiotic use behavior in childhood and adolescence was acquired from the teleinterviews for cases and controls. The participants were asked the same question “During childhood or adolescence, did you receive long‐term or recurrent courses (three or more per year) of antibiotics?”. The characteristics of the case group were selected from the MMC digital medical record system, including age, sex, BMI, comorbidities, drinking frequency, smoking frequency, and family history of diabetes. To make a better comparison, we collected the same baseline information through the teleinterviews for the control group.

### 2.3 Statistical Analysis

We hypothesized that self‐reported LRAU in early life increases the risk of type 2 diabetes. In the UK Biobank cohort, person‐years were calculated from enrollment to the date of the first diagnosis, date of death, or censoring, whichever occurred first. Multivariable Cox proportional hazards regression analyses were performed using age as the time scale. Hazard ratios (HRs) and 95% confidence intervals (CIs) were evaluated for the risk of type 2 diabetes. To measure the effect and assess the potential risks, we calculated the risk difference (RD) and number needed to harm (NNH) [26]. In the crude model, the analysis was adjusted for age. Model 1 adjusted for covariates, including BMI, demographic factors (age, sex, ethnicity, IMD), lifestyle factors (physical activity, drinking frequency, smoking frequency, fruit/meat intake), and family history of diabetes, as they are traditional risk factors for diabetes [27]. Model 2 additionally included adjustment for comorbidities and medication use to control for potential confounding.

To examine potential interaction effects, subgroup analyses were performed according to age, sex, BMI, family history of diabetes, and drinking frequency. We conducted several sensitivity analyses to validate the robustness of our findings. First, we conducted a propensity score matching analysis and overlap propensity score weighting analysis to control the influence of potential confounding factors. Second, we conducted the analysis with complete data without missing values in covariates. Third, we performed a nested case–control study with a logistic regression model, which included all type 2 diabetes cases that occurred during adulthood to minimize possible outcome misclassification.

In the matched case–control trial, unconditional logistic regression models were used to obtain an odd ratio (OR) of type 2 diabetes associated with LRAU in childhood and adolescence [28]. Antibiotic use behaviors exhibit notable disparities between urban and rural areas [29]. Rural regions have been particularly vulnerable to overuse or misuse challenges due to the lack of knowledge and skills among caregivers and limited healthcare access [30]. We matched the diabetes cases and controls for age categories (≤ 35, 35–55, 55–65, ≥ 65) and living location in early life (urban or rural area). We adjusted the OR for matching factors. Other possible confounders evaluated included sex, BMI, alcohol consumption frequency, smoking frequency, and family history of diabetes. Subgroup analyses were also conducted to examine potential interaction effects among age, sex, BMI, family history, and drinking frequency.

For the hypothesis test, a two‐tailed *p* < 0.05 was considered statistically significant. All analyses were performed using R software, version 2022.02.0.

## 3 Results

### 3.1 UK Biobank Cohort

The baseline characteristics of the UK Biobank cohort are presented in Table 1. Compared to nonusers, long‐term or recurrent antibiotic users in early life had a higher proportion of females, a higher BMI, and a higher proportion of family history of diabetes. During the 1 840 944 person‐years of follow‐up in the UK Biobank, 4314 incident cases of type 2 diabetes were identified. Seven hundred seventeen cases came from long‐term or recurrent antibiotic users, and 3597 cases came from non‐long‐term or recurrent antibiotic users. The annual incidence of type 2 diabetes was 2.69/1000 person‐years in long‐term or recurrent antibiotic users and 2.28/1000 person‐years in nonusers.

**TABLE 1:** Baseline characteristics of study participants.

Characteristic	UK Biobank		Case–control study	
	Participants without LRAU	Participants with LRAU	Cases	Controls
Count, *n*	125 676	21 334	263	526
Age, years, mean (SD)^a^	56.02 (7.74)	53.56 (7.44)	51.2 (10.4)	50.1 (11.5)
Age category, *n* (%)^a^				
25–55	55 164 (43.9)	12 441 (58.3)	162 (61.6)	324 (61.6)
55–65	56 912 (45.3)	7803 (36.6)	91 (34.6)	181 (34.4)
> 65	13 600 (10.8)	1090 (5.1)	10 (3.8)	21 (4.0)
Sex, *n* (%)				
Female	68 325 (54.4)	14 987 (70.2)	85 (32.3)	309 (58.7)
Male	57 351 (45.6)	6347 (29.8)	178 (67.7)	217 (41.3)
BMI, kg/m^2^, mean (SD)	26.54 (4.33)	27.06 (4.91)	25.5 (3.9)	24.9 (6.4)
Family history of diabetes, *n* (%)	24 366 (19.4)	4640 (21.7)	132 (50.2)	101 (19.2)
Comorbidities, *n* (%)				
Hypertension	66 719 (53.1)	10 406 (48.8)	75 (28.5)	75 (14.3)
Hyperlipidemia	57 318 (45.6)	9583 (44.9)	59 (22.4)	43 (8.2)
CVD	4961 (3.9)	799 (3.7)	6 (2.3)	17 (3.2)
Alcohol consumption, *n* (%)				
Daily or almost daily	30 300 (24.1)	4281 (20.1)	0 (0)	4 (0.8)
1–4 times a week	64 964 (51.7)	10 590 (49.6)	22 (8.4)	25 (4.8)
1–3 times a month	13 212 (10.5)	2721 (12.8)	86 (32.7)	82 (15.6)
Special occasions/never	17 200 (13.7)	3742 (17.5)	155 (58.9)	415 (78.9)
Smoking status, *n* (%)				
Current	8779 (7.0)	1758 (8.2)	119 (45.2)	111 (21.1)
Previous	43 261 (34.4)	7335 (34.4)	123 (46.8)	380 (72.2)
Never	73 636 (58.6)	12 241 (57.4)	21 (8.0)	35 (6.7)
Living location, *n* (%)^b^				
Urban area	/	/	165 (62.7)	331 (62.9)
Rural area	/	/	98 (37.3)	195 (37.1)

Abbreviations: BMI, body mass index; CVD, cardiovascular disease; LRAU, long‐term or recurrent antibiotic use.

^a^ Age at index date in case–control study and age at baseline in UK Biobank data.

^b^ The living location in the early life for the cases and controls in the case–control study; UK Biobank did not have detailed information on the living location.

Table 2 presents the association between LRAU in early life and the risk of type 2 diabetes within the prospective cohort. A positive association between antibiotic use and the risk of diabetes was observed. In the age‐adjusted model, participants with LRAU had a 37% higher risk of type 2 diabetes than controls (HR, 1.37; 95% CI, 1.26–1.48). After adjusting for demographic factors, including BMI, lifestyle factors, and family history of diabetes in Model 1, the association remained statistically significant but was attenuated (HR, 1.26; 95% CI, 1.16–1.37). Additional adjustments for comorbidities and medication use in Model 2 did not change the estimated effect (HR, 1.26; 95% CI, 1.16–1.37). The absolute risk of antibiotic usage increased by 1.81 per 1000 person‐years for type 2 diabetes (RD, 1.81; 95% CI, 1.42, 2.04). Furthermore, for every 552 individuals exposed to LRAU during their childhood or adolescence, one additional case of diabetes may occur as a result.

**TABLE 2:** Associations between long‐term or recurrent antibiotic use in early life and the risk of type 2 diabetes in the UK Biobank.

Variable	Participants without LRAU	Participants with LRAU
Diabetes cases (*n*)	3597	717
Person‐years of follow‐up	1 574 572	266 372
Incidence rate, *n*/1000 person‐years	2.28	2.69
Age‐adjusted model^a^, HR [95% CI]	1.00 [Reference]	1.37 [1.26, 1.48]
Multivariable adjusted model 1^b^, HR [95% CI]	1.00 [Reference]	1.26 [1.16, 1.37]
Multivariable adjusted model 2^c^, HR [95% CI]	1.00 [Reference]	1.26 [1.16, 1.37]
RD^a^ 1000 [95% CI]	1.81 [1.42, 2.04]	
NNH [95% CI]	552.1 [490.2, 704.9]	

Abbreviations: LRAU, long‐term or recurrent antibiotic use; NNH, number needed to harm.; RD, risk difference.

^a^ Age‐adjusted model: Age was stratified into three categories (35–55, 55–65, ≥ 65).

^b^ Multivariable adjusted model 1: additionally adjusted for sex (female or male), ethnicity (white or non‐white), quintile of the index of multiple deprivations (0,1st, 2nd, 3rd, 4th), BMI, physical activity (low, moderate, high), drinking frequency (daily or almost daily, 1–4 times a week, 1–3 times a month, special occasions only or never), smoking frequency (current, previous, or never), fruit intake (≥ 5 portions or < 5 portions), red and processed meat intake (1 time per week, 2–3 times per week, 3–4 times per week and ≥ 4.0 times per week), and the history of family diabetes (yes or no).

^c^ Multivariable adjusted model 2: based on model 1, additionally adjusted for cardiovascular disease (yes or no), hypertension (yes or no), hyperlipidemia (yes or no), statin use (yes or no), aspirin use (yes or no), non‐steroidal anti‐inflammatory drug use (yes or no), and corticoid use (yes or no).

**FIGURE 2:**
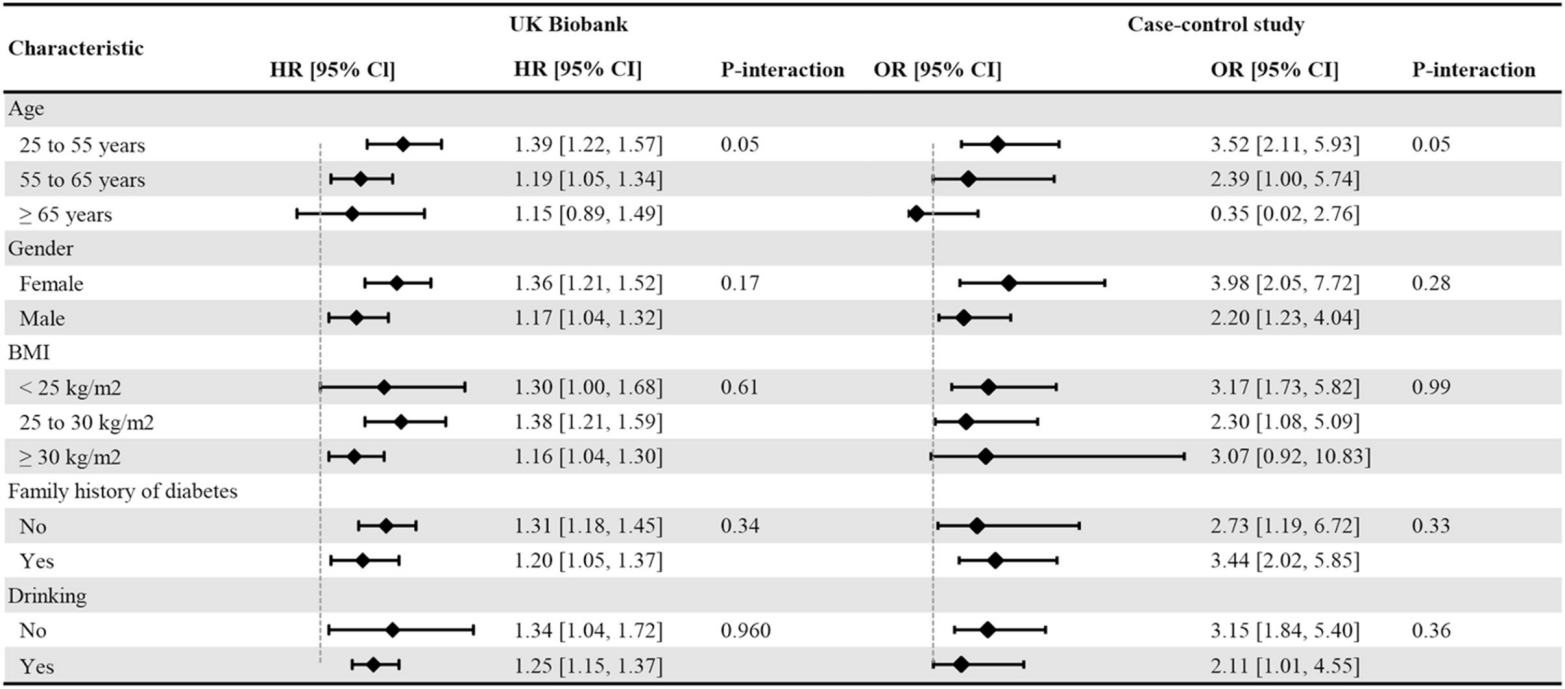
Subgroup analyses of long‐term or recurrent antibiotic use in early life and the risk of type 2 diabetes in the UK Biobank and the case–control study.

Subgroup analyses showed no interaction effects in the risk of type 2 diabetes associated with LRAU based on age, sex, BMI, family history of diabetes, and drinking frequency (Figure 2). For the sensitivity analyses, a positive association was found using a 1:4 propensity score matching analysis (HR, 1.37; 95% CI, 1.26–1.49) and an overlap propensity score weighting analysis (HR, 1.37; 95% CI, 1.26–1.49). After excluding the participants with missing covariate data, the HR in the multivariable Cox model was 1.26 (95% CI, 1.16–1.37), see Table S1. In the logistic regression model, the association between LRAU in early life and type 2 diabetes risk remained stable (adjusted OR, 1.21; 95% CI, 1.11–1.32), see Table S2.

### 3.2 Case–Control Study

The characteristics of the population in the case–control study can be found in Table 1. The case group had a higher proportion of males and a family history of diabetes. The diabetes group also exhibited higher rates of comorbidities (hypertension, hyperlipidemia) and higher rates of smoking and drinking. Among the 263 cases of type 2 diabetes, we have identified 56 participants with a self‐reported history of LRAU during early life. In contrast, out of the 526 healthy participants, only 47 individuals reported a history of LRAU. After adjusting for the matched factors (age and living location), an increased risk of type 2 diabetes (OR, 2.84; 95% CI, 1.86–4.36) was observed in individuals with self‐reported LRAU compared to participants who did not report LRAU in early life. Further adjustment for sex, BMI, family history of diabetes, comorbidities, smoking and drinking still revealed an increased risk of type 2 diabetes for long‐term or recurrent antibiotic users (OR, 3.32; 95% CI, 2.06–5.38), see Table 3. There were no interaction effects on the risk of type 2 diabetes associated with LRAU in early life based on age, sex, BMI, family history of diabetes, or alcohol consumption frequency (Figure 2).

**TABLE 3:** Risk of type 2 diabetes in people who reported LRAU in early life compared to participants who did not report LRAU in the case–control study.

Self‐reported LRAU	Cases	Controls	Age and location‐adjusted model^a^	Multivariable adjusted model^b^
			OR (95% CI)	OR (95% CI)
No	207	479	1.00 [Reference]	1.00 [Reference]
Yes	56	47	2.84 [1.86, 4.36]	3.32 [2.06, 5.38]

Abbreviation: LRAU, long‐term or recurrent antibiotic use.

^a^ Age and living location‐adjusted model is adjusted for age categories (≤ 35, 35–55, 55–65, ≥ 65), living location in early life (urban or rural area).

^b^ Multivariable adjusted model is additionally adjusted for BMI, family history of diabetes (yes or no), cardiovascular disease (yes or no), hypertension (yes or no), hyperlipidemia (yes or no), drinking or smoking (yes or no).

## 4 Discussion

In this prospective cohort study and case‐control study, we found that LRAU in early life was associated with an increased risk of type 2 diabetes. This association remained consistent after adjusting for traditional diabetes risk factors.

The consistent findings from previous studies provide evidence of the relationship between antibiotic use and the increased risk of type 2 diabetes. One study conducted by our group previously reported an increased risk of type 2 diabetes (HR, 1.20) associated with LRAU over the past 4 years in women from NHS and NHS II cohorts [16]. National studies from Denmark (*n* = 5 600 000), Finland (*n* = 26 883) and Korea (*n* = 201 459) also showed an increased risk for type 2 diabetes in participants with LRAU [31, 32, 33]. Moreover, our findings conducted on the UK and Chinese populations persisted with a previous nested case–control study based on a large UK database which revealed a dose–response effect between antibiotic course and diabetes risk (OR, 1.37) [34]. Our finding was also consistent with another Chinese population‐based cross‐sectional study that demonstrated an association between antibiotic exposure from the environment and the increased risk of type 2 diabetes [15].

In this study, we first observed that LRUA in early life has a profound effect on the development of type 2 diabetes. Our findings align with emerging epidemiological evidence that has already suggested a potential association between early antibiotic exposure in children and later pediatric obesity [35, 36]. For instance, a prospective cohort study that included infants born into the Military Health System database revealed a link between antibiotic use in early childhood and the diagnosis of childhood obesity [36]. Furthermore, a meta‐analysis of 14 observational studies demonstrated a 21% higher risk of obesity development in children exposed to antibiotics [37]. These findings collectively suggest that childhood obesity, known to cause inflammation, may play a pivotal role in mediating the relationship between obesity and insulin resistance, ultimately leading to the development of type 2 diabetes [38].

The potential mechanism underlying the increased risk of type 2 diabetes with antibiotic use remains unclear. One potential mechanism may involve the perturbation of the gut microbiota, as antibiotics have a profound effect on gut microbial diversity and composition [20]. The gut microbiota contains a pool of antibiotic resistance genes (ARGs), and antibiotic treatment increases the pool of resistance genes in the gut [39]. Studies have shown that patients with type 2 diabetes exhibit gut bacterial dysbiosis and that ARG features are associated with an increased risk of type 2 diabetes [6, 40]. Animal studies have also demonstrated that antibiotic treatment alters intestinal microbiota, reduces tissue inflammation, improves insulin signaling, and enhances glucose metabolism in mice prone to obesity and diabetes [13].

Our study has several strengths. We conducted prospective analyses based on data from the UK Biobank to investigate the effect of LRAU in early life. Subsequently, we designed a matched case–control study to validate the association in the Chinese population, thereby enhancing the generalizability of our research findings across populations with diverse genetic backgrounds. Importantly, to our knowledge, our study is one of few epidemiological studies focused on LRAU in early life and the risk of type 2 diabetes. Moreover, we took great care to control for traditional diabetes risk factors and carefully adjusted the effect size for potential confounding variables. In the case–control study, we sourced diabetes cases from MMC, affording us the advantage of accessing comprehensive and detailed information on baseline characteristics.

However, our study also has certain limitations that should be acknowledged. In the Chinese population, the implementation of the Internet Health Management System was relatively delayed, hindering our ability to access prescription records for participants' antibiotic use during childhood or adolescence. Consequently, we had to rely on self‐reported information gathered through teleinterviews. This method of data collection may introduce recall bias, as participants may not accurately remember their past antibiotic usage habits. Furthermore, the definition of LRUA as recurrent courses (three or more times in 1 year) made it difficult to determine the precise timing of antibiotic use and precluded further dose–response analysis. Previous studies have suggested that different classes of antibiotics may have varying effects on the increased risk of diabetes [33, 34], but our data from the UK Biobank and the teleinterviews did not provide detailed information on the specific classes of antibiotics used. Therefore, future studies should aim to collect more accurate data on antibiotic use habits in early life and investigate their associations with type 2 diabetes in larger prospective populations.

## Author Contributions

All authors were involved in drafting or revising the article, and all authors approved the final version to be published. Study conception and design: Zijun Li, Qiangsheng He, Jinqiu Yuan, Kehu Yang. Accessed and collected data: Songbo Fu, Xiaoping Sun, Jinqiu Yuan. Analyzed data: Zijun Li, Xin Xing, Jinyu Zhao, Mina Ma, Jinqiu Yuan. Writing draft: Zijun Li, Qiangsheng He, Kehu Yang. Editing draft: Kehu Yang, Danni Wang, Jinqiu Yuan. Zijun Li and Kehu Yang are the guarantors of this work and, as such, have full access to all the data in the study and take responsibility for the integrity of the data and the accuracy of the data analysis.

## Conflicts of Interest

The authors declare no conflicts of interest.

## Supporting information


**Data S1.** Supporting Information.

## Data Availability

Reasonable requests to access the data from the Chinese Metabolic Management Center used in this study may be sent to the corresponding authors. Data from UK Biobank are available on application at www.ukbiobank.ac.uk/register‐apply.
