# Supplementary material for: Long‐Term or Recurrent Antibiotic Use in Early Life and the Risk of Type 2 Diabetes: A Population‐Based Prospective Cohort and a Case–Control Study
Source: J Diabetes. 2025 Jun 18;17(6):e70113. doi: 10.1111/1753-0407.70113 (PMC12176498; doi:10.1111/1753-0407.70113)
Supplement: Supplementary file 1 — Data S1. Supporting Information. [file JDB-17-e70113-s001.docx]

**Supplementary Materials**

**Supplementary Table 1** Sensitivity analyses of long-term or recurrence antibiotic use in early life and the risk of type 2 diabetes in the UK Biobank

| **Sensitivity analyses methods** | **Cases/ Person-years** | **HR [95%CI]** |
| --- | --- | --- |
| **Using propensity score matching analysis**^*^ | | |
| Non-users | 2071/1014268.2 | 1.00[Reference] |
| Long-term antibiotic user | 717/266345.5 | 1.37 [1.26, 1.49] |
| **Using overlap propensity score weighting analysis** ^†^ | | |
| Non-users | 2071/1574572.4 | 1.00[Reference] |
| Long-term antibiotic user | 717/266371.6 | 1.37 [1.26, 1.49] |
| **Excluding the participants with missing covariate data** | | |
| Non-users | 2837/1293104.5 | 1.00[Reference] |
| Long-term antibiotic user | 559/216140.3 | 1.26 [1.16,1.37] |

^*^Long-term antibiotic users and non-users in early life were matched using a 1:4 nearest neighbour calliper matching method without replacement based on propensity scores. Covariates used to estimate the propensity score included age, gender, ethnicity, BMI, family history of type 2 diabetes, smoking status, alcohol consumption, physical activity, fruit and vegetable intake, and comorbidities.

^†^Estimated effects were based on the overlap-weighted Cox model. The propensity score was derived by multivariate logistic regression conditional on baseline covariates. Multivariable-adjusted Cox model was fitted with adjustment of age, gender, ethnicity, BMI, family history of type 2 diabetes, smoking status, alcohol consumption, physical activity, fruit and vegetable intake, and comorbidities.

**Supplementary Table 2** Sensitivity analysis of long-term or recurrence antibiotic use in early life and the risk of type 2 diabetes with logistic regression model in the UK Biobank.

| **LRAU** | **Cases** | **Controls** | **Crude model^*^** |  | **Multivariable adjusted model^†^** |
| --- | --- | --- | --- | --- | --- |
|  |  |  | **OR (95% CI)** |  | **OR (95% CI)** |
| No | 3597/4314 | 122079/142696 | 1.00 [Reference] |  | 1.00 [Reference] |
| Yes | 717/4314 | 20617/142696 | 1.49 [1.37, 1.62] |  | 1.21 [1.11, 1.32] |

LRAU, long-term or recurrence antibiotic use

^*^Crude model is adjusted for sex (male or female), and age categories (≤35, 35-55, 55-65, ≥65).

^†^Multivariable adjusted model is additionally adjusted for ethnicity (white or non-white), quintile of the index of multiple deprivations (0,1st, 2nd, 3rd, 4th), BMI, physical activity (low, moderate, high), drinking frequency (daily or almost daily, 1-4 times a week, 1-3 times a month, special occasions only or never), smoking frequency (current, previous, or never), fruit intake ((≥ portions or <5 portions), red and processed meat intake (1 time per week, 2-3 times per week, 3-4 times per week and ≥4.0 times per week), the history of family diabetes (yes or no), systolic blood pressure, cardiovascular disease (yes or no), hypertension (yes or no), hyperlipidemia (yes or no).
